# What Did We Learn from Our Cochlear Implant Revisions?

**DOI:** 10.22038/ijorl.2025.77740.3612

**Published:** 2025

**Authors:** Cigdem Kalaycik Ertugay, Ozgur Yigit, Ecem Sevim Akı

**Affiliations:** 1 *Department of Otorhinolaryngology/Head and Neck Surgery, Kozyatagı Acıbadem Hospital, Istanbul, Turkey. *; 2 *Department of Otorhinolaryngology/Head and Neck Surgery, University of Health Sciences Istanbul Education and Research Hospital, Istanbul, Turkey. *; 3 *Department of Otorhinolaryngology/Head and Neck Surgery, Dr.Şinasi Can (Kadıkoy) Acıbadem Hospital, Istanbul, Turkey.*

**Keywords:** Cochlear implants, Cochlear implantation, Implantable hearing aids, Hearing loss, Sensorineural hearing loss

## Abstract

**Introduction:**

We aimed to report our clinic’s 11-year experience with cochlear implant (CI) revision surgeries.

**Materials and Methods:**

This was a retrospective observational study. Patients who underwent CI and revision surgery at the same tertiary institution were enrolled in the study. Patients whose primary surgery was performed at another institution were excluded from the study. The patients’ clinical charts, surgical records, and audiological and oral language outcomes were retrospectively examined.

**Results:**

Thirty-three (29 children, 4 adults) of 720 patients (871 CI) at our clinic required revision surgery, representing a revision surgery rate of 4.58%. The most common reason for revision was device failure (10 patients), followed by skin and electrode problems, with electrode tip fold-over in 6 patients, a broken electrode cable in 1 patient, skin flap complications in 6 patients, displacement of the magnet in 1 patient, cholesteatoma in 1 patient, electrode migration in 6 patients, misplacement of the electrode array into the internal acoustic canal in 1 patient, and explantation of the electrode cable in the external auditory canal in 1 patient. We had only one major complication after revision surgery.

**Conclusion:**

We recommend performing routine postoperative imaging, even if intraoperative telemetries are normal, to diagnose electrode misplacement or electrode tip fold-over. Additionally, we recommend long-term regular follow-up of children, in particular, because our study showed that the number of revision surgeries was higher in children who received implants at an early age.

## Introduction

Cochlear implantation (CI) is an alternative treatment method for patients with severe sensorineural hearing loss (SNHL) who do not benefit sufficiently from conventional hearing aids in terms of word and sentence recognition. However, CI is not completely free of risk, and some patients may require revision surgeries. 

Hochmair-Desoyer and Burian first reported on CI revision surgery in 1985 (1). Re-implantation may be needed because of device failure (hardware or software failure), skin flap complications, electrode migration, misplacement of the electrode array, and electrode tip fold-over, among other reasons. Although surgeon-related complications have decreased in recent years, others, such as device failure, still present challenges. 

Additionally, in children who are very young and cannot speak, parents may not notice problems with the device. Furthermore, revision surgeries have several risks, such as intracochlear trauma, decreased electrode activation, and decreased speech perception. Although revision surgery does not routinely result in these complications, and several reports evaluating the safety of this procedure have been conducted, updated studies are still needed (2-4). 

In recent years, revision surgery has become more important because the number of clinicians and centers performing CI has increased worldwide. At the same time, as electrode technology has improved, more modern surgical techniques (hearing preservation) have been created. In light of this situation, surgeons should know their experiences after revision surgeries performed in their own clinics, the expected failure rates and auditory performance of patients, and they should advise patients on this issue.

We aimed to report our 11-year experience with CI revision surgeries and review our clinic’s cochlear re-implantation data in both adults and children, with a particular focus on the reasons for revision, surgical challenges, and functional outcomes.

## Materials and Methods

This retrospective observational study was approved by the local ethics committee of the University of Health Sciences Istanbul Education and Research Hospital. Severe to profound sensorineural hearing loss was confirmed preoperatively in all our patients by auditory brainstem response or pure tone audiometry testing. Preoperative computed tomography and inner ear magnetic resonance images of all patients were reviewed to determine abnormalities in the inner ear and vestibulocochlear nerve. 

All patients were assessed by experienced speech-language therapists and psychologists before and after surgery to establish their main communication mode. 

Young children were administered the Infant-Toddler Meaningful Auditory Integration Scale (MAIS), and older children were administered the Early Speech Perception test or the MAIS. All CI decisions were made by a council of otolaryngologists, audiologists, radiologists, speech-language therapists, and psychologists. All primary and revision surgeries were performed by the same senior surgeon. Routine plain X-rays were taken in all patients on the first postoperative day to assess appropriate electrode placement. All patients were invited for at least 1-year follow-up with 2-month intervals between visits.

The clinical charts, primary surgical and revision surgical records, audiological outcomes (such as pure tone average thresholds), and oral language characteristics of all patients with bilateral severe to profound SNHL who underwent CI and revision surgery at the same tertiary institution between 2008 and 2019 were retrospectively examined.

The following data were recorded: demographic data, risk factors for hearing loss, period of hearing loss, age at primary implantation, age at revision surgery, gender, imaging findings, surgical findings, the cause of revision surgeries, audiological outcomes, and oral language characteristics before and at least one year after revision surgeries. 

We divided the patients according to their age upon primary implantation, with the patients who first received implants before 18 years of age classified as the child group and those older than 18 as the adult group. Post-auricular incisions were made, and a subperiostal pocket technique was used for implant fixation in the primary surgeries. Since 2018, the surgeon has also drilled a groove for the electrode cable between the internal unit and mastoid cavity when using a Med-El implant. 

Care was taken to leave a distance of at least 1.5 cm between the edge of the receiver and the incision. Afterwards, a cortical mastoidectomy and posterior tympanotomy were performed. Slow electrode insertion through a round window was used in almost all cases. 

If the round window niche was not visible, endoscope-assisted CI or direct electrode insertion through the external ear canal was performed. 

Furthermore, if any anatomic variation hindered cortical mastoidectomy and posterior tympanotomy, direct electrode insertion through the external ear canal was preferred. 

If the round window niche was still not visible, despite these surgical techniques, a cochleostomy was made. Steroid injection through the round window or cochleostomy was performed just before electrode insertion. Radiologic evaluation was performed in all cases one day postoperatively, in addition to intraoperative auditory monitoring. 

The implant manufacturers used were Med-El, Cochlear, Advanced Bionics, and Oticon. Statistical analysis was performed using IBM® SPSS 17.0 software (SPSS Corp.; Armonk, NY, USA). 

Quantitative variables are expressed as means or medians, and ordinal variables are expressed as sample size (%). In the analysis of qualitative independent data, children and adults were compared using the Pearson Chi-square test. A P value of less than 0.05 was considered statistically significant.

## Results

We performed a total of 871 CI during the 11-year study period. Five hundred and sixty-nine patients (79%) received a unilateral cochlear implant. Bilateral CI has been covered by insurance since December 2016 for children aged less than 4 years in the country where the study was conducted. 

After this time, 151 patients (21%) received bilateral CI (a total of 720 patients). Of these 151 patients, 41 (27.2%) received the implants simultaneously and 110 (72.8%) received them sequentially. Thirty-three (29 children, 4 adults) of 720 patients (4.58%) required revision surgery (3.78 % of 871 CI) (Table 1).

**Table 1:** Patient data regarding the number of primary and revision surgeries and age of patients. (CI: Cochlear implant) (n: Number)

	**Children, n = 29**	**Adults, n = 4**
Number of revision CI surgeries (%)	29 (4.84% of children) (4.02% of total patients)	4 (3.72% of adults), (0.56% of total patients)
Mean age of the primary CI surgery	3.1 (1–17.3)	37.65 (18–69)
Mean age of revision CI surgery	5.33 (1.6–9)	44.25 (22–60)

Of the 29 children, 9 (31%) were male and 20 (69%) were female. Twenty-eight children had prelingual hearing loss, and 1 child, who also had a cochlear anomaly, had perilingual hearing loss. The mean ages of the primary and revision surgeries were 4.04 years (ranging from 1.1–16.3 years) and 5.33 years (ranging from 1.6–16.5 years), respectively. 

Of the 4 adults, 2 (50%) were male and 2 (50%) were female. The mean ages of the primary and revision surgeries were 37.65 years and 44.25 years, respectively (Table 1). Regarding hearing loss etiology, 5 children and 1 adult had cochlear anomalies. Two children had bilateral Mondini deformity, 1 child had contralateral cochlear nerve aplasia in addition to bilateral Mondini deformity, 1 child had bilateral cochlear hypoplasia, and another child had bilateral incomplete partition (IP) of the cochlea type 3.

One adult had cochlear hypoplasia in the implanted side and contralateral Michel deformity. 

Three adults had undergone chronic otitis media (COM) surgery before CI. The other risk factors for hearing loss of all patients are presented in Table 2. 

**Table 2:** Data of patients undergoing revision CI surgery according to risk factors for hearing loss. (CI: Cochlear implant) (n: Number)

	**Children, n = 29**	**Adults, n = 4**
No risk factor (%)	4 (13.79%)	0 (0%)
Prematurity (%)	5 (17.24%)	0 (0%)
Neonatal intensive care unit stay (%)	3 (10.34%)	1 ( 25%)
Consanguinity (%)	12 (41.37%)	1 (25%)
Cytomegalovirus infection (%)	1 (3.44%)	0 (0%)
Family history of hearing loss (%)	10 (34.48%)	0 (0%)
Ototoxic medication during pregnancy (%)	3 (10.34%)	0 (0%)
Chronic otitis media (%)	0 (0%)	3 (75%)
Waardenburg (%)	1 (3.44%)	0 (0%)
Inner ear anomalies (%)	5 (17.24%)	1 (25%)

**Fig 1:**
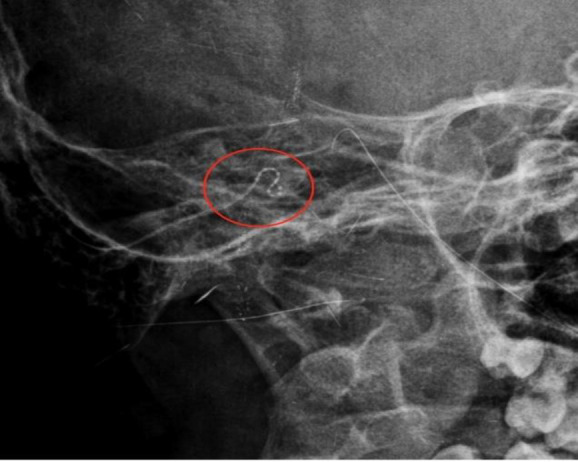
X-ray of misplacement of the electrode array in the internal acoustic canal

The causes of revision surgery were device failure in 10 patients (8 Med-El, 1 Cochlear, 1 Oticon), electrode tip fold-over in 6 patients (3 Med-El, 2 Cochlear, 1 Advanced Bionics), broken electrode cable due to trauma in 1 patient (1 Cochlear), skin flap complications in 6 patients (4 Cochlear, 2 Advanced Bionics), displacement of the magnet in 1 patient (1 Cochlear), tympanic membrane perforation and cholesteatoma in 1 patient (1 Med-El), electrode migration in 6 patients (4 Med-El, 2 Cochlear), misplacement of the electrode array into the internal acoustic canal in 1 patient (1 Med-El) (Figure 1), and explantation of the electrode cable in the external auditory canal of 1 patient (1 Med-El) (Table 3).

**Table 3:** Patient data regarding causes of revision surgeries. (CI: Cochlear implant) (n: Number)

	**Children, n = 29**	**Adults, n = 4**
Device failure (%)	10 (1.67% of children) (1.38% of total patients) (1.14% of total implants)	0 (0%)
Electrode tip fold-over (%)	5 (0.83% of children) (0.69% of total patients) (0.57% of total implants)	1 (0.81% of adults) (0.14% of total patients) (0.11% of total implants)
Broken electrode cable due to trauma (%)	1 (0.17% of children) (0.14% of total patients) (0.11% of total implants)	0 (0%)
Skin flap complications (%)	4 (0.67% of children) (0.55% of total patients) (0.46% of total implants)	2 (1.63% of adults) (0.28% of total patients) (0.23% of total implants)
Displacement of the magnet (%)	1 (0.17% of children) (0.14% of total patients) (0.11% of total implants)	0 (0%)
Tympanic membrane perforation and cholesteatoma (%)	1 (0.17% of children) (0.14% of total patients) (0.11% of total implants)	0 (0%)
Electrode migration (%)	6 (1.01% of children) (0.83% of total patients) (0.69% of total implants)	0 (0%)
Misplacement of the electrode array into the internal acoustic canal (%)	1 (0.17% of children) (0.14% of total patients) (0.11% of total implants)	0 (0%)
Explantation of electrode cable in the external auditory canal (%)	0 (0%)	1 (0.81% of adults) (0.14% of total patients) (0.11% of total implants)

Device failure, which occurred within 4 years of primary implantation, was observed during routine follow-up in pediatric implanted patients with prelingual deafness. Three of the 10 patients had a history of trauma. The age of patients upon first implantation ranged from 1 to 4 years old. Four patients had undergone unilateral CI, whereas 6 patients had undergone bilateral simultaneous CI. No intraoperative complications were observed in the revision surgeries of these patients, although two had bilateral Mondini deformity. There was no significant difference in audiometric data post-operatively in comparison to best pre-failure data. Pure tone average (0.5–4 kHz) was between 25 and 40 dB before and 1 year after the surgeries (p < 0.001). Additionally, oral language characteristics did not worsen after revision surgeries. 

Skin flap complications were observed in 4 pediatric patients within 1 year of primary implantation. The age of patients upon first implantation ranged from 1 to 3 years. Three of these patients had undergone unilateral CI, whereas 1 had undergone simultaneous bilateral CI. We had to explant the internal unit, leaving the electrode array inside the cochlea and performing CI on the contralateral side in 3 pediatric patients with unilateral implants. We performed re-implantation on the primary side in 2 of these patients after a 2-year follow-up period, but the parents of 1 patient did not accept re-implantation on the complicated side. The fourth pediatric patient, who had undergone simultaneous bilateral CI at 1 year old, had a skin flap infection on the left side.

The infection did not improve, and skin flap necrosis occurred despite several medical therapies and surgical drainages. Although the surgeon performed rotational flap surgery, skin flap necrosis recurred. Therefore, the internal unit had to be removed, leaving the electrode array inside the cochlea. The patient was followed up for 4 years, and he uses his right implant routinely. His parents did not accept re-implantation because the development of his language skills was better than that of his twin, who had also undergone bilateral simultaneous CI. There was no significant difference in audiometric data post-operatively in comparison to best pre-failure data (p < 0.001). 

Additionally, oral language characteristics did not worsen. Furthermore, 2 adult patients with COM who underwent mastoidectomy before CI had skin flap necrosis. Skin flap complications occurred 1 year and 3 years after primary implantation, respectively. The surgeon drilled an implant bed and relocated the internal unit posteroinferiorly; afterwards, a rotational flap was transposed to cover the internal unit, and there was no need to explant the implant during the 5 years follow-up period in these 2 adult patients. 

The patient in whom the electrode array was misplaced in the internal acoustic canal had IP type 3 anomaly, and no significant complications were observed during revision surgery other than perilymphatic gusher. Interoperative imaging was used in revision surgery to avoid misplacement of the electrode array again. The cholesteatoma was observed in 1 patient who had undergone primary CI surgery when she was 6 years old. At 5 and 7 years after primary Cl surgery, she received two revision surgeries because of cholesteatoma. 

Explantation of the electrode cable in the external auditory canal was observed in 1 patient. She was admitted to the clinic at 19 years old with a history of perilingual hearing loss. She had cochlear hypoplasia in the right side of the ear and contralateral Michel aplasia. 

Furthermore, the sigmoid sinus was located very anteriorly on the right side, hindering cortical mastoidectomy and the posterior tympanotomy technique; therefore, direct electrode insertion through the external ear canal was performed. Three years after primary implantation, she was admitted with the complaint of decreased benefit of hearing capability from the implantation. The electrode was partially explanted, and the electrode cable was visible in the external auditory canal. Therefore, revision surgery was performed without any complications.

Only one major complication occurred after revision surgery. The electrode array was misplaced in the vestibule in the revision surgery of a girl although she had no inner ear anomaly. The reason for her revision surgery was electrode migration. The misplacement of the electrode array was observed in radiography when she stated that she felt a problem with the implant during routine follow-up. She had undergone primary CI surgery when she was 6 years old, and the electrode migration was observed 1 year after primary surgery. 

Two minor complications occurred after revision surgeries. CI was performed with cul de sac closure of the ear canal in an adult patient because of his previous COM surgery. However, he had also undergone revision surgery because of the electrode tip fold-over. Two weeks after the revision surgery, the sutures of the cul de sac opened spontaneously.

The other patient was a 16-year-old boy with IP of the cochlea type 3 anomaly. He had undergone revision surgery because of the misplacement of the electrode array in the internal acoustic canal. Six months after revision surgery, we examined tympanic membrane perforation and performed myringoplasty. 

## Discussion

The rate at which patients underwent revision surgery in the department where this study was conducted (CI failure rate) was 4.58 % (29 children, 4 adults). Our data was consistent with the literature (Cote: 5.4%, Amaral: 4.3%, Brown:5.5%) (5-7). The most common reason for revision was device failure, followed by skin and electrode problems. Although revision surgery is a difficult challenge, language and auditory performances were not affected in most cases, possibly because of the early detection of problems through routine follow-up at short intervals after primary surgery. 

Device failure has been stated as one of the common causes of revision surgery in the literature (7,8,9,10). Kim et al. showed that the most common cause of revision surgery is device failure, with a rate of 65% (9). In the present study, device failure was found in 30.3% (10/33) of revision cases and was the most common reason for revision surgery.

Cullen et al. found a history of head trauma in 40% of patients with device failure (11). Gosapath et al. showed that device failure is more common in children than adults, and they attributed this to children being more prone to head trauma (12). In the present study, all device failures were observed in children, and a history of head trauma was present in 30% of these cases. Interestingly, the rates of device failure due to internal device failure and head trauma were both lower than comparable percentages reported in the literature. This indicates that the rate of technical failure for other reasons was higher in the present study. 

However, the reason for this is unknown. The diagnosis of device failure is sometimes difficult if the reason is soft failure because the results of integrity tests are normal in such circumstances. Although health insurance limits follow-up to 2 years in Turkey, we think this follow-up period is inadequate because device failure was detected within 4 years of primary implantation in this study, and most of these patients had no complaint. 

Rates of major skin flap–related complications have been reported to range from 1.8–8.2% in the literature (13,14). In the present study, major flap-related complications were observed in 0.83% of all patients and 18.2% of revision patients. Low et al. and Garcia-Valdecasas et al. did not find a significant relationship between age and the frequency of flap necrosis in their study . Ikeya et al. stated that a previous history of COM may lead to flap-related complications after cochlear implant surgery (15-17). In the present study, we observed the rate of flap-related complications to be higher in adults (50%) than children (13.8%), but this study had a low number of patients. This situation may be attributable to a history of surgery due to COM before CI in these patients. Therefore, if a patient has a history of COM surgery, we suggest that a more postero-inferior location of the internal unit should be used than in standard surgeries and that an implant bed should be drilled. Moreover, we observed that in 50% of pediatric patients, the posterior part of the internal unit was not located subperiosteally, which may have caused the skin flap infections. 

The surgeon has made some changes in his surgical technique in response to this complication in recent years. For example, since 2018, a groove has been drilled for the electrode cable between the internal unit and mastoid cavity when using Med-El implants. 

Zuniga et al. and Grolman et al. found an incidence of electrode tip fold-over in CI of 1.98% and 5.6%, respectively (18,19). Lassig et al. showed that this was the cause of 13% of revision surgeries (20). In our study, electrode tip fold-over was found in 0.83% of patients who underwent cochlear implant surgery. Also, electrode tip fold-over was the reason for reoperation in 18.2% of patients who underwent revision cochlear implant surgery.

Similar to the studies in the literature, we observed normal telemetric measurements intraoperatively in all patients in the present study. Routine x-rays were performed on day one postoperatively even if intraoperative telemetric measurements were normal. This practice was changed after the experience with tip fold-over. 

Now, if there is any uneasiness during electrode insertion or any problem in intraoperative telemetric measurements, we perform a postoperative x-ray before waking the patient to enable any fold-over to be fixed. 

Minor head trauma has been shown to be the most common factor causing displacement of the magnet in the literature, and it has been stated that displacement of the magnet might occur more commonly in young males (21). 

Yun et al. showed that thinness of the scalp and curvature of the skull may play a role in the displacement of the magnet, in addition to head trauma (22). 

In our study, displacement of the magnet was observed in a 3-year-old boy who had no history of head trauma, only pain in the magnet area. Minor head trauma had not been noticed by the family, and thin skin may have caused magnet displacement in this case. The prevalence of cholesteatoma after CI ranges from 4.8–12.2 % (23). 

Generally, it is seen in patients with a history of COM or previous ear surgery, and cholesteatoma formation related to CI is very rare (24). It has been stated in the literature that the development of cholesteatoma in post-implanted patients who had no history of COM might be the result of damage to and thinning of the external ear canal and tympanic membrane during CI (25). Only 1 patient had a cholesteatoma after CI in the present study, and she underwent two operations as a result of cholesteatoma after implantation. This patient’s primary CI surgery was performed in the clinic’s first year of surgery. 

Intraoperative imaging was not performed in the primary surgery of the patient with IP of the cochlea type 3 because no problem occurred during surgery, which may have been an error. A limitation of the present study is its small size. This is due to its retrospective nature and the fact that patients who underwent primary surgery in another institution were not included; therefore, the number of patients who underwent revision surgeries was small.

## Conclusion

This study retrospectively reported revision cochlear cases to analyze the reasons for revision in a tertiary hospital. The greatest strength of the present study is that all the primary and revision surgeries were performed by the same senior surgeon, who made several modifications to his surgical technique over the study period in response to the revision experience. The clinic’s revision surgery rate was 4.58%, and the most common reason for revision was device failure, followed by skin and electrode problems. Revision surgery can be needed due to problems with the patient or the implant, and patients who require revision may vary in age. When we discussed the rate and type of complications on a yearly basis, the complications associated with surgical technique, such as cholestatoma formation and skin flap problems, were found to have decreased. However, the rates of other reasons, such as device failure, have not changed in recent years. Therefore, we suggest that every clinician should inform the patient and family about the possible causes of revision and their own clinic’s revision causes and rates.

## References

[1] Hochmair-Desoyer I, Burian K (1985). Reimplantation of a molded scala tympani electrode: Impact on psychophysical and speech discrimination abilities. Ann. Otol. Rhinol. Laryngol..

[2] Rivas A, Marlowe AL, Chinnici JE, Niparko JK, Francis HW (2008). Revision cochlear implantation surgery in adults: Indications and results. Otol. Neurotol..

[3] Balkany TJ, Hodges AV, Gómez-Marín O, Bird PA, Dolan-Ash S, Butts S et al (1999). Cochlear reimplantation. Laryngoscope.

[4] Henson AM, Slattery WH 3rd, Luxford WM, Mills DM (1999). Cochlear implant performance after reimplantation: A multicenter study. Am. J. Otol..

[5] Côté M, Ferron P, Bergeron F, Bussiere R (2007). Cochlear reimplantation: Causes of failure, outcomes and audiologic performance. Laryngoscope.

[6] Amaral MSAD, Reis ACMB, Massuda ET, Hyppolito MA (2019). Cochlear implant revision surgeries in children. Braz. J. Otorhinolaryngol..

[7] Brown KD, Connell SS, Balkany TJ, Eshraghi AE, Telischi FF, Angeli SA (2009). Incidence and indications for revision cochlear implant surgery in adults and children. Laryngoscope.

[8] Jiang Y, Gu P, Li B, Gao X, Sun B, Song Y (2017). Analysis and management of complications in a cohort of 1,065 minimally invasive cochlear implantations. Otol. Neurotol..

[9] Kim SY, Kim MB, Chung WH, Cho YS, Hong SH, Moon IJ (2020). Evaluating reasons for revision surgery and device failure rates in patients who underwent cochlear implantation surgery. JAMA Otolaryngol. Head Neck Surg..

[10] Rayamajhi P, Kurkure R, Castellino A, Kumar S, Ha M, Nandhan R (2021). A clinical profile of revision cochlear implant surgery: MERF experience. Cochlear Implants Int..

[11] Cullen RD, Fayad JN, Luxford WM, Buchman CA (2008). Revision cochlear implant surgery in children. Otol. Neurotol..

[12] Gosepath J, Lippert K, Keilmann A, Mann WJ (2009). Analysis of fifty-six cochlear implant device failures. ORL J. Otorhinolaryngol. Relat. Spec..

[13] Davids T, Ramsden JD, Gordon KA, James AL, Papsin BC (2009). Soft tissue complications after small incision pediatric cochlear implantation Laryngoscope.

[14] Hopfenspirger MT, Levine SC, Rimell FL (2007). Infectious complications in pediatric cochlear implants. Laryngoscope.

[15] Low WK, Rangabashyam M, Wang F (2014). Management of major post-cochlear implant wound infections Eur. Arch. Otorhinolaryngol..

[16] Garcia-Valdecasas J, Jimenez-Moleon JJ, Sainz M, Fornieles C, Ballesteros JM (2009). Prophylactic effect of clarithromycin in skin flap complications in cochlear implants surgery. Laryngoscope.

[17] Ikeya J, Kawano A, Nishiyama N, Kawaguchi S, Hagiwara A, Suzuki M (2013). Long-term complications after cochlear implantation. Auris. Nasus. Larynx..

[18] Zuniga MG, Rivas A, Hedley-Williams A, Gifford RH, Dwyer R, Dawant BM et al (2017). Tip fold-over in cochlear implantation: Case series. Otol. Neurotol..

[19] Grolman W, Maat A, Verdam F, Simis Y, Carelsen B, Freling N et al (2009). Spread of excitation measurements for the detection of electrode array foldovers: A prospective study comparing 3-dimensional rotational x-ray and intraoperative spread of excitation measurements. Otol. Neurotol..

[20] Lassig AA, Zwolan TA, Telian SA (2005). Cochlear implant failures and revision. Otol. Neurotol..

[21] Nichani JR, BroomWeld SJ, Saeed SR (2004). Displacement of the magnet of a cochlear implant receiver stimulator package following minor head trauma. Cochlear Implants Int..

[22] Yun JM, Colburn MW, Antonelli PJ (2005). Cochlear implant magnet displacement with minor head trauma. Otolaryngol. Head Neck Surg..

[23] Halawani R, Aldhafeeri A, Alajlan S, Alzhrani F (2019). Complications of post-cochlear implantation in 1027 adults and children. Ann. Saudi. Med..

[24] Kaila R, Evans RA (2005). Cochlear implant infection due to cholesteatoma. Cochlear Implants Int..

[25] Bort A, Portmann D, Guindi S (2015). Cholesteatoma presenting as a late complication of cochlear implant surgery: Case report and literature review. Rev. Laryngol. Otol. Rhinol. (Bord).

